# Natural bioactive substances for the control of food-borne viruses and contaminants in food

**DOI:** 10.1186/s43014-020-00040-y

**Published:** 2020-11-30

**Authors:** Yao Pan, Zeyuan Deng, Fereidoon Shahidi

**Affiliations:** 1 Department of Biochemistry, Memorial University of Newfoundland, St. John’s, NL A1B 3X9 Canada; 2 State Key Laboratory of Food Science and Technology, University of Nanchang, Nanchang, 330047 Jiangxi China

**Keywords:** Food-borne virus, Food contamination, Natural bioactive substances, Food safety

## Abstract

**Abstract:**

Food-borne viruses and contaminants, as an important global food safety problem, are caused by chemical, microbiological, zoonotic, and other risk factors that represent a health hazard. Natural bioactive substances, originating from plants, animals, or microorganisms, might offer the possibility of preventing and controlling food-borne diseases. In this contribution, the common bioactive substances such as polyphenols, essential oils, proteins, and polysaccharides which are effective in the prevention and treatment of food-borne viruses and contaminants are discussed. Meanwhile, the preventive effects of natural bioactive substances and the possible mechanisms involved in food protection are discussed and detailed. The application and potential effects of natural bioactive substances in the adjuvant treatment for food-borne diseases is also described.

**Graphical abstract:**

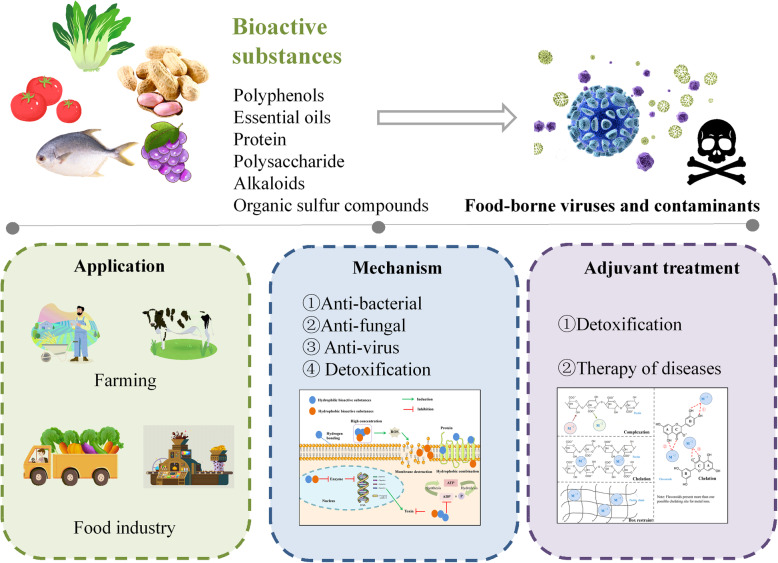

## Introduction

Viruses and some contaminants may cause food-borne diseases and these have become a hot issue in the field of food science and public health (Li et al. 2013). In recent years, food safety problems arising from food-borne viruses and contaminants have become a common global concern (European Food Safety Authority and European Centre for Disease Prevention and Control 2018). Researchers believe that food-borne viruses are often transmitted through the fecal-oral, water, and person-to-person contact route, or caused by contaminated foods, such as marine pollution that contaminates shellfish and fish (Ethelberg et al. 2010; Zomer et al. 2010).

Several measures such as good production chains (avoiding fecal), strict control of water contamination on farming and irrigation, and good public health awareness can, to some extent, prevent food-borne viruses. Physical and chemical control methods have been widely used in food production (Baert et al. 2009; Li et al. 2009). However, there is little research on the prevention and control of food-borne viruses and contaminants in food by natural bioactive substances. This review briefly describes the formation of food-borne viruses and contaminants in food, the action mechanism of natural bioactive substances to control them, and their recent use in the food industry.

### Definition of food-borne viruses and contaminants in food

Viruses are very small infectious microorganisms composed of a DNA or RNA genome enclosed within a protein coat. Food-borne viruses, as obligate intracellular parasites, depending on the living host to survive, are globally recognized as the major causes of nonbacterial gastroenteritis or respiratory problems (Nelluri and Thota 2018). Many viruses show high resistance to stressors such as heat, drying, freezing, and UV light, among others, and may survive for long periods in food or the body (Newell et al. 2010). Common food-borne viruses include human noroviruses (HNoV), hepatitis A virus (HAV), hepatitis E virus (HEV), rotavirus (RV), Aichi virus (AiV), astroviruses, sap viruses, adenoviruses serotypes 40 and 41, coxsackievirus A and B, parvoviruses, and other enteroviruses and picornaviruses (Souza 2015).

Food contaminants refer to harmful substances that compromise the safety or quality of food and may cause diseases. Food contamination may occur due to the presence of toxicants (phytotoxins), bacterial contamination (*Salmonella)*, pesticide residues, and physical and chemical hazards produced during food processing such as the generation of polycyclic aromatic hydrocarbons, among others (Kuswandi et al. 2017).

### Defects of chemical/physical control measures on food-borne diseases

Most food-borne diseases are infections caused by a variety of food-borne viruses, and harmful toxins or chemicals that have contaminated food. For example, according to a report from Food and Drug Administration (FDA) (Foodborne illness-causing organisms in the U.S. What you need to know 2018), unpasteurized fruit or vegetable juices may contain *E. coli O157*, *Salmonella*, and *Clostridium botulinum*. Some raw fish and shellfish contain norovirus, hepatitis A, and many other pathogens. Therefore, with the new developments in food science and technology, many methods for the prevention and treatment of food-borne diseases have emerged. The traditional methods of physical and chemical control have some shortcomings and hence particular attention should be paid to novel methods to make certain that the safety of food is ensured. However, it should be noted that some of these methods may still suffer from certain drawbacks and could render deleterious effects such as: (1) Reduced the nutritional value of food. For example, high-pressure processing shows antimicrobial effects on orange juice, while it decreases the content of vitamin C significantly after processing (Bull et al. 2004). (2) The residue of harmful substances may also remain in the food. For example, sodium hypochlorite and peroxyacetic acid prevent cross-contamination during the washing process by causing a reduction in the number of pathogens present in lettuce, but the residues of sodium hypochlorite and peroxyacetic acid remain in the food (Baert et al. 2009). (3) Viruses cannot be completely inactivated. For instance, high-pressure processing changes the protein structures of viruses but not for RNA on murine norovirus-1 in oysters (Li et al. 2009).

Therefore, there is more and more interest in using natural bioactive substances in the farming and production of food for the prevention and control of food-borne viruses and contaminants. This demand by consumers is leading the search for the development of alternative natural substances that can simultaneously extend the shelf life of food while providing a high degree of safety regarding food-borne diseases.

## Bioactive substances for the control of food-borne viruses and contaminants

In recent years, research on controlling food-borne viruses and contaminants in food has caused widespread concern. Among them, research on natural bioactive substances has focused mainly on different aspects as detailed below.

### Polyphenols

Polyphenols, including phenolic acids, stilbenes, flavonoids, and lignans, among others, have multiple functions for human health such as neuroprotective, cardioprotective, antioxidant, and anticarcinogenic effects (Zhang and Tsao 2016). Polyphenols are widely found in fruits and vegetables, herbs, grains, and other plant foods (Abbas et al. 2017).

It is known that some polyphenols are resistant to food-borne viruses. Functional components on roots of *Glycyrrhiza uralensis*, including glyasperin, glycyrin, 2′-methoxyisoliquiritigenin, licoflavonol, and glyasperin D, have proven to be resistant to rotaviruses (Kwon et al. 2010). The extracts of black raspberry which contain gallic acid, caffeic acid, ellagic acid, quercetin, and cyanidin-3-glucoside, have been shown to exert a negative effect on human norovirus (Zhang et al. 2012). In general, the possible mechanism of polyphenols anti-virus effect can be summarized as follows. (1) Decreasing the virus replication. For example, previous research has indicated that potato peel (containing gallic acid, chlorogenic acid, caffeic acid, ferulic acid, rutin, and quercetin) inhibits human norovirus via down-regulating the replication of viruses (Table 1) (Silva-beltrán et al. 2017). (2) Binding with virus receptors. For instance, tannic acid extracted from Chinese *gall pomegranate* inhibited the noroviruses via binding with the histoblood group antigens (HBGAs) as receptors, thus preventing the virus from entering the host cells (Table 1) (Zhang et al. 2012). (3) Causing structural damage to the virus. In this connection, an earlier study suggested that aged-green tea extract inactivated the virus by causing structural damage (Table 1) (Falcó et al. 2019).

**Table 1:** Research on natural bioactive substances and effects on food-borne viruses and contaminants

Bioactive substances	Functional component	Source	Effect	Possible mechanism	Reference
Polyphenols	Glyasperin, Glycyrin 2′-Methoxyisoliquiritigenin Licoflavono, Glyasperin D	Roots of *Glycyrrhiza uralensis*	↓Group A rotaviruses	↓Virus absorption to cells ↓Viral replication after entry	Kwon et al. (2010)
	Tannic acid	Chinese Gall Pomegranate	↓Noroviruses	↓NoV P proteins binding to their HBGA receptors	Zhang et al. (2012)
	Caffeic acid, Cyanidin-3-rutinoside, 3,4-Dihydroxybenzoic acid, Rutin	Mulberry	Human norovirus	↓Viral replication	Oh et al. (2013)
	Gallic acid, Caffeic acid, Ellagic acid, Quercetin, Cyanidin-3-glucoside	Black raspberry	↓Human norovirus	↓Viral gene expression ↓Plaque formation	Lee et al. (2016)
	Gallic acid, Chlorogenic acid, Caffeic acid, Ferulic acid, Rutin, Quercetin	Potato peel	↓Human Enteric Viruses	↓Viral replication	Silva-beltrán et al. (2017)
	Epigallocatechin gallate	Green tea	↓Murine norovirus ↓Hepatitis A virus	Nonspecific binding to viral surface proteins ↓Viral attachment to cell membrane receptors	Randazzo et al. (2017)
	Aged-green tea extract	*Camellia sinensis L.*	↓Human norovirus	↓Binding of virus to histo-blood group antigens structural damage	Falcó et al. (2019)
	Pinosylvin	Wood	↓Gram-negative/ positive bacteria	Interacting with cell membrane	(Plumed-ferrer et al. 2013)
	Tea polyphenols extract	Green tea	↓*Staphylococcus aureus* ↓Salmonella serotype	Affecting the formation of the cell membrane	(Hongmei Zhang et al. 2014)
	Ellagic acid, Gallic acid, Rutin	*Passiflora ligularis* Juss. fruit	↓*fungal strains Candida albicans* ↓Aspergillus niger	structural or functional damage to the bacterial cell membrane	(Saravanan and Parimelazhagan 2014)
	Epicatechin	Green tea	↓Acrylamide	Trapping of carbonyl compounds ↓ lipid oxidation	(Liu et al. 2015)
	Proanthocyanidins	Grape seed	↓Residual nitrite	↓Oxidation	(Wang et al. 2015)
Essential oil	Carvacrol, Thymol methyl ether	*Zataria multiflora Boiss*	↓Norovirus	Inactivating the virus	(Elizaquível et al. 2013)
	Carvacrol	Oregano oil	↓Murine norovirus	Binding to the virus ↓Virus adsorption to host cells	(Gilling et al. 2014)
	Ocimene, a-Terpinolene Citral, d-Limonene	Lemongrass essential oil	↓Norovirus	↓Viral replication	(Kim et al. 2017)
	Limonene, β-Pinene, γ-Terpinene, Cineole, ɑ-Pinene, Camphor, Camphene	Lemon, sweet orange, Grapefruit, rosemary cineole	↓Hepatitis A Virus	Inactivating the virus	(Battistini et al. 2019)
	Piperitone, α-Phellandrene, *p*-Cymene	Australian *Eucalyptus*	↓Gram-negative/ positive bacteria	Interacting with cell membrane	(Gilles et al. 2010)
	Carvacrol	Herbs	↓*S. aureus* *Staphylococcus epidermidis*	Interacting with cell membrane	(Miranda-novales and Solo 2012)
	Geraniol	Herbs	↓Gram-negative bacteria	Interacting with cell membrane	(Miranda-novales and Solo 2012)
	Cinnamaldehyde	Cinnamon	↓*E. coli* and S. aureus	Change Membrane potential	(Zhang et al. 2016a, b)
	Methyl cinnamate γ-terpinene	*Ocimum gratissimum*	↓Aflatoxin B1	↓Aflatoxin secretion	(Prakash et al. 2011)
	Cymene	*Cuminum cyminum* (L.) seed	↓Aflatoxin B1	↓Aflatoxin secretion	(Kedia et al. 2014)
Protein	Lactadherin	Human and Bovine Milk	↓Rotavirus	Affect protein structure	(Petersen et al. 2004)
	Lactoferrin	Breast milk	↓Hepatitis A Virus	Interfering with virus-receptor Interaction	(Waarts et al. 2005)
	Lactadherin	Human and Bovine Milk	↓Poliovirus	↓Viral replication	(Pan et al. 2006)
	Lactadherin	Breast milk	↓Murine norovirus	↓Viral replication	(Ishikawa et al. 2013)
	α-Caseins	Milk	↓Gram-positive bacteria	Cationic glycopeptides	(Benkerroum 2010)
	Hepcidin TH1–5	Fish	↓Gram-positive bacteria	↓Activity	(Najafian and Babji 2012a)
Polysaccharides	Chitosan	Crustaceans	↓Human noroviruses	↓Viral replication	(Davis et al. 2012)
	Water-soluble Chitosan	Crustaceans	Enteric viruses	Viral structural damage	(Davis et al. 2015)
	Extract from *Houttuynia cordata*	Houttuynia cordata	↓Murine norovirus ↓Human noroviruses	Deforming and inflating virus particles	(Cheng et al. 2019)
	Polysaccharide Streptomyces virginia H03	*Streptomyces virginia* H03	*Staphylococcus aureus* *Listeria monocytogenes* *Escherichia coli*	Affecting cytoplasmic membrane permeability /DNA binding	(He et al. 2010)
	Sulfated polysaccharides	Gray triggerfish	↓Gram-negative/ positive bacteria	Interacting with cell membrane	(Krichen et al. 2015)
	Polysaccharides extract	Algae	↓ Escherichia coli	↓proliferation	(Rivas et al. 2017)
	Polysaccharides extract	Algae	↓ Salmonella spp.	↓proliferation	(Rivas et al. 2017)
	Chitosan	Crab processing discards	↓Ion contaminants	Metal chelation	(Gamage and Shahidi 2007)
Alkaloids	Pelleteriene	Pomegranate seed	↓Staphylococcus aureus	↓membrane permeability	(Ismail et al. 2012)
	Pyrazinecarboxamide derivative Indole derivative	Alkaloid derivative	↓Hepatitis A Virus ↓Norovirus	↓replication of the virus	(Hwu et al. 2017)
	Quinine	The bark of the cinchona	↓Malaria Possible↓COVID-19	↓replication of the virus	(Achan et al. 2011) (Gautret et al. 2020)
	Alkaloid extracts	*Solanum nigrum*	↓*Escherichia coli,* ↓*Proteus mirabilis,* ↓*Staphylococcus aureus,* ↓*Pseudomonas aerogenosa*	Interact with cell membrane	(Jasim et al. 2015)
Organic sulfur compounds	Sulfur compounds extracts	Fresh garlic by-products	↓*S. aureus,* ↓*S. enteritidis,* ↓*E. coli, B. cereus,* ↓*L. monocytogens*	Interact with cell membrane	(Jang et al. 2018)
	Sulfur compounds extracts	Green vegetables	↓Hepatitis A Virus ↓Norovirus	↓replication of the virus	(Sofy et al. 2018)
	Sulfur compounds extracts	Herbs	↓*Bacillus cereus,* ↓*Campylobacter jejuni,* ↓*Clostridium, Escherichia coli,* ↓*Listeria* ↓*Monocytogenes,* ↓*Salmonella enterica,* ↓*Staphylococcus*	Interact with cell membrane	(Ikeura and Koabayashi 2015)
	Diallyl sulfides, Diallyl monosulfide, Diallyl disulfide, Diallyl trisulfide, Diallyl tetrasulfide	Chive oil	*Staphylococcus aureus* *Listeria monocytogenes* *Escherichia coli*	Interact with cell membrane	(Rattanachaikunsopon and Phumkhachorn 2008)
	Allicin	Garlic	possible↓virus	↑Immunity	(Rahman 2007)

Polyphenols are also known to control both biological and chemical contaminants in food, such as anti-bacterial, anti-fungal, and other chemical hazards (Table 1). Their antibacterial action is usually achieved by affecting the properties of the bacterial cell membrane (Plumed-Ferrer et al. 2013; Zhang et al. 2014). Meanwhile, polyphenols, such as ellagic acid, gallic acid, and rutin can also show an anti-fungal effect by affecting cell membrane morphology (Saravanan and Parimelazhagan 2014). Moreover, polyphenols show inhibition for chemical contaminants. For example, epicatechin reduces the acrylamide content of food by trapping of carbonyl compounds and decreasing lipid oxidation (Liu et al. 2015). Proanthocyanidins could down-regulate the residual nitrite because of their antioxidant effects (Wang et al. 2015). Those polyphenols may affect the nitrite depletion by affecting pH in the food matrix or antioxidant properties. When the pH of meat is lower than 6.0, nitrite can be transformed into nitrous acid or nitric oxide, which can react with polyphenols or other endogenous substances (Viuda-Martos et al. 2010).

### Essential oils

Essential oils (EOs) (esp. herb-based essential oils), which are enriched in plant-derived volatile aromatic compounds, have potential as natural agents for food preservation because of their antibacterial, antifungal, and antioxidative activities and have long been applied as flavoring agents in food (Gilling et al. 2014). EOs play an important role in food processing due to the above-mentioned myriad of characteristics. Some EOs have antiviral effects due to the inhibition of virus replication, anti-absorption of the virus, and inactivation (Table 1). For example, carvacrol and thymol methyl ether from *Zataria multiflora Boiss* were suggested to inhibit norovirus via an inactivation mechanism. Meanwhile, carvacrol from oregano oil could bind to the virus and inhibit virus adsorption to host cells (Gilling et al. 2014). Meanwhile, lemongrass essential oil has been suggested to inhibit norovirus by reducing the virus’s replication (Kim et al. 2017). Besides, some EOs also display anti-bacterial effects and are used in the food industry and food packaging in recent years (Table 1**)** (Wen et al. 2016). EOs from Australian *Eucalyptus* (containing piperitone, α-phellandrene, *p*-cymene) could interact with the cell membrane of gram-negative and gram-positive bacteria (Gilles et al. 2010). Carvacrol and geraniol from herb oils have been shown to inhibit *S. aureus* and some gram-negative bacteria via interacting with their cell membrane, respectively (Miranda-novales and Solo 2012). Furthermore, cinnamon oil (cinnamaldehyde as the main component) could change the membrane potential (a difference in electric potential between the interior and the exterior of a biological cell) of *E. coli* and *S. aureus* (Zhang et al. 2016a, b) The essential oil also showed anti-fungal properties in some fruits. For instance, mustard and clove essential oil combinations in the vapor phase synergistically inhibited *B. cinerea* in strawberries (Aguilar-González et al. 2015). Moreover, methyl cinnamate, γ-terpinene, and cymene reduced the aflatoxin secretion in food products (Prakash et al. 2011; Kedia et al. 2014).

### Protein

Numerous studies have demonstrated that many proteins, especially those from cow milk, breast milk, and fish, can effectively inhibit food-borne viruses, and bacteria (Table 1) (Li et al. 2013). Milk contains an array of proteins such as casein, lactoferrin, alpha-lactalbumin, and beta-lactoglobulin with useful bioactivities and antiviral activities (Petersen et al. 2004). Lactadherin, which is widely found in cow milk and breast milk, has proven to show anti-virus activity via affecting viruses’ protein structure or reducing viral replication (Petersen et al. 2004; Pan et al. 2006; Ishikawa et al. 2013). α-Caseins from milk were also down-regulated gram-positive bacteria via their cationic glycopeptides (Benkerroum 2010). Moreover, some fish proteins, such as hepcidin TH1–5 was found to inhibit the activity of gram-positive bacteria (Najafian et al. 2012b).

### Polysaccharide

Polysaccharides, composed of monosaccharide units bound together by glycosidic bonds, are polymeric molecules of carbohydrates (Ferreira et al. 2015). Some polysaccharides exert anti-viral activity by inhibiting viral entry into host cells (Table 1).

Chitosan, a biopolymer produced by the deacetylation of chitin derived from the exoskeleton of crustaceans, is one of the most widely used materials in this field (Davis et al. 2012). Previous research has found that water-soluble chitosan could inhibit enteric viruses by interfering with viral replication or damaging the structure of viruses (Davis et al. 2012; Davis et al. 2015). Chitosan extracted from crab processing discards decreased the ion contaminants in water via metal chelation (Gamage and Shahidi 2007).

Algal polysaccharides are obtained from algae and may include high amounts of mucopolysaccharides, as well as storage and cell wall structure polysaccharides. Some polysaccharides extracted from algae exert proliferation effects on *Escherichia coli* and *Salmonella spp.* (Rivas et al. 2017), which showed antimicrobial potential against pathogenic and spoilage microorganisms in food.

Besides, polysaccharides from some herbs, for example, *Houttuynia cordata*, could inhibit murine norovirus and human noroviruses via deforming and inflating virus particles (Cheng et al. 2019). Meanwhile, polysaccharides extracts from fish skin could interact with the cell membrane of gram-negative and gram-positive bacteria (Krichen et al. 2015), thus inhibiting their growth and are being used to extend the shelf life of food.

### Alkaloids

Alkaloids are abundant in herbal extracts and are one of the most common plant-based formulations in traditional Chinese medicine (Zheng et al. 2018). Despite the serious health impact of alkaloids, they are also used in the control of some foodborne viruses (Prasad et al. 2020). For example, some alkaloids derivatives (eg., pyrazinecarboxamide derivatives, and indole derivatives) have been developed as drugs to protect against viruses such as norovirus in Japan and Russia by interfering with the replication of the virus (Hwu et al. 2017). Moreover, traditional plant-based medicine for treating SARS coronavirus (SARS-CoV) was developed in the Guangdong Province of China in 2002–2003. For example, quinine, an alkaloid, has been used in the treatment of malaria since the 1960s (Achan et al. 2011). Moreover, a structural analog of quinine was found to be effective in reducing the viral load in SARS-CoV-2 (COVID-19) (Gautret et al. 2020). Alkaloids are also used as basic medical agents because of their analgesic and antibacterial properties (Sharaibi and Osuntogun 2014). For instance, alkaloid compounds of *Solanum nigrum* were proven to inhibit the growth of *Escherichia coli*, *Proteus mirabilis, Staphylococcus aureus, Pseudomonas aerogenosa* (Jasim et al. 2015)*.* Pelleteriene, an alkaloid from pomegranate seed, was found to be effective in preventing the growth of *Staphylococcus aureus* by affecting the structure of cell membrane (Ismail et al. 2012).

### Organic sulfur compounds

Organic sulfides are widely found in spices such as garlic, scallions, and onions as well as some cruciferous vegetables (e.g., kale, mustard leaf, and broccoli) (Goncharov et al. 2016). Several studies have shown that they exhibit antimicrobial activity (Table 1). For example, sulfur compounds extracted from fresh garlic by-products show significant antimicrobial activity against *S. aureus*, *S. enteritidis*, *E. coli*, *B. cereus*, and *L. monocytogens* (Jang et al. 2018). Organic sulfur compounds extracts from herbs are known to have antimicrobial properties against various bacteria such as *Bacillus cereus*, *Campylobacter jejuni*, *Clostridium*, *Escherichia coli*, *Listeria monocytogenes*, *Salmonella enterica*, *Staphylococcus* (Ikeura and Koabayashi 2015). Both diallyl sulfides, diallyl monosulfide, diallyl disulfide, diallyl trisulfide, and diallyl tetrasulfide extracted from chive oil showed antimicrobial activities (Rattanachaikunsopon and Phumkhachorn 2008). Although few research on the antiviral properties of organic sulfides has been carried out, some organic sulfur compounds found in some green vegetables may show protection against viruses (eg., hepatitis A virus, and norovirus) (Sofy et al. 2018). Besides, some sulfides have been shown to enhance immunity by rendering the anti-viral effect. For example, allicin, one of the major organosulfur compounds in garlic was found to improve immune function and to avoid a viral attack (Rahman 2007).

### Other compounds

There are still other natural bioactive substances that show anti-viral effects. For example, the saponin extracts from *Eucalyptus citriodora* have been reported to possess antivirus activity (Zhou et al. 2014). Saponins from *Medicago sativa* were found to show antimicrobial activity against gram-positive and gram-negative bacteria (Avato et al. 2006). Antimicrobial activities were also found in other Saponin-rich extracts from plants, such as guar, quillaja, yucca, and soybean (Hassan et al. 2010). Moreover, vitamin K could be used as a green biocide with high bactericidal efficacy toward both *Escherichia coli* and *Listeria innocua* after seven times repeated daylight exposure (Zhang et al. 2019). Vitamin D was also proven to innate immunity by regulating the production of antimicrobial peptides and cytokine response, which show its potential for use as antimicrobial drugs (Youssef et al. 2011). A detailed discussion about these bioactive compounds is beyond the scope of this review and needs to be reviewed separately.

## Application of bioactive substances in food production

Contaminants may enter food through the farming environment (eg., air, feed/soil, water) or during food processing (Fischer et al. 2016). The application of bioactive substances for controlling food contaminants and food-borne viruses during farming and processing in the food industry is discussed and detailed in the following subsections.

### Farming

#### Planting

Heavy metals/benzodiazepines in the soil, and overuse of most synthetic pesticides/fungicides during planting has created different types of environmental and toxicological problems. On the one hand, phytoremediation can remove, sequester, or stabilize many organic and inorganic contaminants, including heavy metals, and reduce benzodiazepines in the soil (Kidd et al. 2015). Although natural bioactive substances are not added directly to the soil in this strategy, it still utilizes the bioactive substances (e.g. organic acids, polyphenols) in plants in response to the chelation of metal ions and the reaction of contaminants after absorption (Thakur et al. 2016).

On the other hand, the popularity of natural bioactive pesticides is once again increasing and some plant products are being used globally as green pesticides (Cantrell et al. 2012). For example, some plant extracts, containing polyphenols, gums, resins, and essential oils have already been used as antimicrobial substances against a wide array of microorganisms (Gurjar et al. 2012; Zaker 2016). Some plant extracts show antimicrobial effect and serve as plant defense mechanisms against pathogenic microorganisms, such as inhibiting the chitin synthase enzyme of fungi (Cantrell et al. 2012).

#### Animal husbandry

The process of animal breeding is very easy to be infected by a wide range of bacteria, thus damaging human health. Natural bioactive substances mainly enhance the antibacterial ability of animals by modifying their diet.

For example, *Salmonella*, one of the human pathogens, is always consumed from raw or undercooked contaminated poultry products. Previous studies have shown that primary production, mixing, and processing, increase the chance of contact with insects, and wild animals which could easily cause contamination at several stages. However, adding natural organic acids into drinking water for animals could greatly reduce post-harvest crop contamination with *Salmonella * (Awad and Ghareeb 2014). Besides, infectious pathogens of birds could be reduced by modifying the ingredients and nutrient composition of their diets (Vandeplas et al. 2010). The mechanism of this strategy may be related to the fact that the diet contains high-fiber and polysaccharide that may modify the microflora and physicochemical balance in the gastrointestinal tract (GIT) of birds, thus improving a bird’s resistance to colonization by *Salmonella* and other pathogens (Vandeplas et al. 2010). Moreover, the feeding of medium-chain fatty acids (C8-C10) could also regulate the GIT of birds and has been shown to reduce the gut colonization of broilers by *Campylobacter* (van Gerwe et al. 2010). The study also used essential oils to prevent or reduce the colonization of broilers by *Campylobacter* in a similar manner (Umaraw et al. 2017). Besides, some natural bioactive substances, such as lysozyme, have been used as growth-promoting subtherapeutic antibiotic in swine feed because of their ability to cleave the peptidoglycan component of bacterial cell walls (Oliver and Wells 2015).

#### Aquaculture industry

Natural bioactive substances are mainly used to control contaminants in aquaculture in two ways. One is by replacing various chemotherapeutic agents with natural bioactive substances to reduce the rate of infection from bacteria as well as the residue and accumulation of harmful agents in organisms. The other one uses bioactive compounds to remove harmful substances such as polycyclic aromatic hydrocarbons, polychlorinated biphenyls, organochlorinated pesticides, potentially toxic elements, and residues of veterinary drugs and antibiotics in aquaculture products (fish, crustaceans, and mollusks).

In this connection, some studies have intensified efforts to exploit natural products such as herbs in developing alternative dietary supplements that enhance growth performance as well as the health and immune system of fish (Syahidah et al. 2015). Thus, the antibacterial potential of aqueous and methanolic extracts of Malaysian local herbs was tested for this reason (Najiah et al. 2011). Meanwhile, according to a previous study (Zilberg et al. 2010), essential oil from rosemary displayed positive results in inhibiting a common tilapia pathogen. These herbs show anti-bacteria/anti-toxicity effects due to the presence of various bioactive substances like alkaloids, flavonoids, phenolic acids, terpenoids, steroids, and essential oils (Citarasu 2010). Besides, some natural bioactive substances (eg. chitosan) could remove the contaminants in water, thus reducing the acquisition of harmful substances by living organisms (Gamage and Shahidi 2007).

### Food industry

Several preservation techniques, such as heat treatment, salting, acidification, and drying have been used in the food industry to extend the shelf life of food by preventing the growth of some microorganisms or food-borne viruses. Furthermore, foods preserved with natural additives have gained wide attention in recent years (Table 2). Such natural bioactive substances can be directly added into the product formulation, spread on the food surface, added into the packaging material, or used in antimicrobial films to maintain their activity for shelf life extension (Lucera et al. 2012).

**Table 2:** Strategies used in the food industry to control food contaminants and food-borne virus through bioactive substances

Strategies	Natural substances	Application	Results	References
Food additives	Essential oil	Yogurt	Anti-microbiological effects	(Singh et al. 2011)
	Clary, Sage, Juniper, Lemon, and Majoram Essential oil	Apple juice	Anti-yeast	(Tserennadmid et al. 2011)
	Oregano essential oil	Apple fruits	Anti-microbiological effects	(Lopez-Reyes et al. 2010)
	Essential oil from *O. vulgare* L. and *Rosmarinus officinalis* L.	Vegetables	Anti-bacterial growth	(De Azeredo et al. 2011)
	Carvacrol and thymol essential oil	Lemon	Antifungal effects	(Pérez-Alfonso et al. 2012)
	Lysozyme	Cheese	Anti-microbiological effects	(Sinigaglia et al. 2008; Quintieri et al. 2012)
	Lactoferrin	Chicken filets	Anti-microbiological effects	(Del Olmo et al. 2012)
	Chitosan	Beef	Anti-microbiological, Anti-virus	(Duran and Kahve 2020)
	Epigallocatechin gallate-polyunsaturated fatty acid esters	Food products	Anti-virus effects	(Shahidi and Zhong 2010)
Coatings	Polysaccharides	Fruits/Vegetables	Anti-microbiological effects	(Aloui and Khwaldia 2016)
	Oregano essential oil and whey protein	Chicken breast	Anti-microbiological effects	(Fernández-Pan et al. 2014)
	Chitosan and pomegranate peel extract	White shrimp	Anti-microbiological effects	(Yuan et al. 2016)
	Oregano and thyme essential oil	Food package	Anti-microbiological effects	(Solano and de Gante 2012)
	Chitosan	Food package	Form a protective layer	(Pinheiro et al. 2012)
Edible films.	Polyphenols from propolis	Food package	Antifungal properties	(Pastor et al. 2010)
	Lysozyme, Lactoferrin	Food package	Anti-microbiological effects	(Barbiroli et al. 2012)
	Chitosan, Essential oil	Food package	Anti-microbiological effects Form a protective layer	(Hafsa et al. 2016; Shahidi and Hossain 2020)

#### Food additives

Most natural bioactive substances are added directly to the food system as additives. These natural products include essential oils from various plants, such as thyme, oregano, cinnamon, clove, and rosemary (Gutierrez et al. 2008; Gutierrez et al. 2009). For example, essential oils are known to control spoilage microorganisms when added into yogurt (Singh et al. 2011). Essential oils (e.g. celery, sage, juniper, lemon, and marjoram essential oil) may also be used to preserve apple juice because of their anti-yeast effects (Tserennadmid et al. 2011). Some of the bioactive substances are obtained from animal sources such as enzymes, including lysozyme, and lactoferrin. For instance, lysozyme and lactoferrin could extend the shelf life of mozzarella cheese through their anti-microbiological effects (Sinigaglia et al. 2008; Quintieri et al. 2012). However, another study suggested that lysozyme only shows significant anti-microbiological effects at high concentrations (Conte et al. 2011). Moreover, polysaccharides (chitosan) and phenolic acid derivatives are used in food preservation for preventing microbial activity, food-borne pathogens, and spoilage bacteria. For example, chitosan coating has proven to render anti-microbiological, anti-virus, and antioxidant effects in beef (Duran and Kahve 2020). In addition, esters of epigallocatechin gallate with polyunsaturated fatty acids exhibited anti-hepatitis C virus (HCV) activity (Shahidi and Zhong 2010).

#### Spreading of coatings on food surface

Food industries have used coatings on highly perishable foods to protect their nutritional properties, extend their shelf life, and reduce the negative effects caused by processing (eg., enzymatic browning, texture breakdown, and off-flavor development) for many years (Sánchez-Ortega et al. 2014). The coating solution could either be highly viscous or non-highly viscous. The highly viscous solution has been used in dipping, one of the coating methods applied for fruits and vegetables, by many food industries. Meanwhile, when the coating solution is not highly viscous, spraying, and bushing would be used for preservation purposes (Valdés et al. 2015). It has been shown that forming an active coating by some bioactive substances on the surface of food can extend their shelf life. For example, spreading essential oils on the food surface has been reported in the literature as a useful technique to improve the quality of products (Andevari and Rezaei 2011). This strategy may use several bioactive substances. For instance, coatings enriched with oregano essential oil combined with whey protein could extend the refrigerated shelf life of chicken breast through the inhibition of microorganisms (Fernández-Pan et al. 2014). Chitosan coating combined with pomegranate peel extract (containing polyphenols) showed antimicrobial effects on white shrimp during iced storage (Yuan et al. 2016). Polysaccharides have also been widely used as coating materials for fresh fruits and vegetables due to their ability as carriers of natural antimicrobial substances to preserve postharvest quality (Aloui and Khwaldia 2016).

It is important to note that the active coatings on the surface of foods could act as semi-permeable membranes, thus reducing gas transfer rates to extend their shelf-life. In addition, some of the coatings consist of proteins and polysaccharides which form cross-linking (process of forming tridimensional networks by linking polymer chains by covalent or non-covalent bonds), hence increasing their water-resistance and barrier properties to avoid food spoilage and contamination (Azeredo and Waldron 2016).

#### Forming edible films

Edible films may be considered as packaging material in which preservative agents serve as thin layers applied to them without being directly added to the food product but would be eaten together with the food (Salgado et al. 2015). These films are biodegradable or renewable products, which can be completely degraded by microorganisms and finally changed into carbon dioxide, water, methane, and some other biomass residues (Reddy et al. 2013). Edible films based on carbohydrates or proteins may contain antimicrobial agents (e.g. lysozyme, chitosan, essential oils) (Irkin and Esmer 2015). For example, hydroxypropyl  methylcellulose based films combined with propolis (containing polyphenols) show physical and antifungal properties (Pastor et al. 2010). In addition, previous results suggest that essential oils (EOs) can be added to the film to improve antimicrobial and antioxidant properties (Shahidi and Hossain 2020).

Several bioactive agents can be incorporated into or onto coatings, such as essential oil, chitosan, and lysozyme. For instance, it has been demonstrated that essential oils of oregano and thyme display anti-fungal effects when incorporated into coatings (Solano and de Gante 2012). κ-Carrageenan and chitosan have proven to be suitable edible coatings that could be used by the food industry (Pinheiro et al. 2012). Therefore, edible films may be considered as combinations consisting of various natural substances. In these, the most important component is the biopolymer, such as proteins (e.g. soybean proteins, wheat gluten, corn zein, sunflower proteins, gelatin, whey, casein, and keratin), lipids (e.g. wax, triacylglycerols, monoacylglycerols, and free fatty acids) and polysaccharides (e.g. cellulose derivatives, starches, alginates, pectins, chitosans, carrageenans, gums, and fibers). These films also contain solvents (eg., water or ethanol) and additives (e.g. antioxidants, antimicrobials, and flavors) (Salgado et al. 2015). Previous studies have shown that a film based on chitosan which contained *Eucalyptus globulus* essential oil rendered antimicrobial effects in food products (Hafsa et al. 2016). Moreover, a variety of combination treatments of some natural bioactive agents, including lysozyme in starch-based edible packaging film showed significant antimicrobial effects (Bhatia and Bharti 2015).

In general, edible films are suggested to decrease the diffusion of active compounds onto food surfaces and maintain their concentrations at a critical level for inhibition of microbial growth during the storage period (Gyawali and Ibrahim 2014). Such films also act as an effective barrier to gas transfer such as oxygen and carbon dioxide, thus inhibiting the growth of microorganisms (Cazón et al. 2017).

## Action mechanisms

### Anti-bacterial and anti-fungal mechanisms

#### Destruction of the cell membrane

**Fig. 1:**
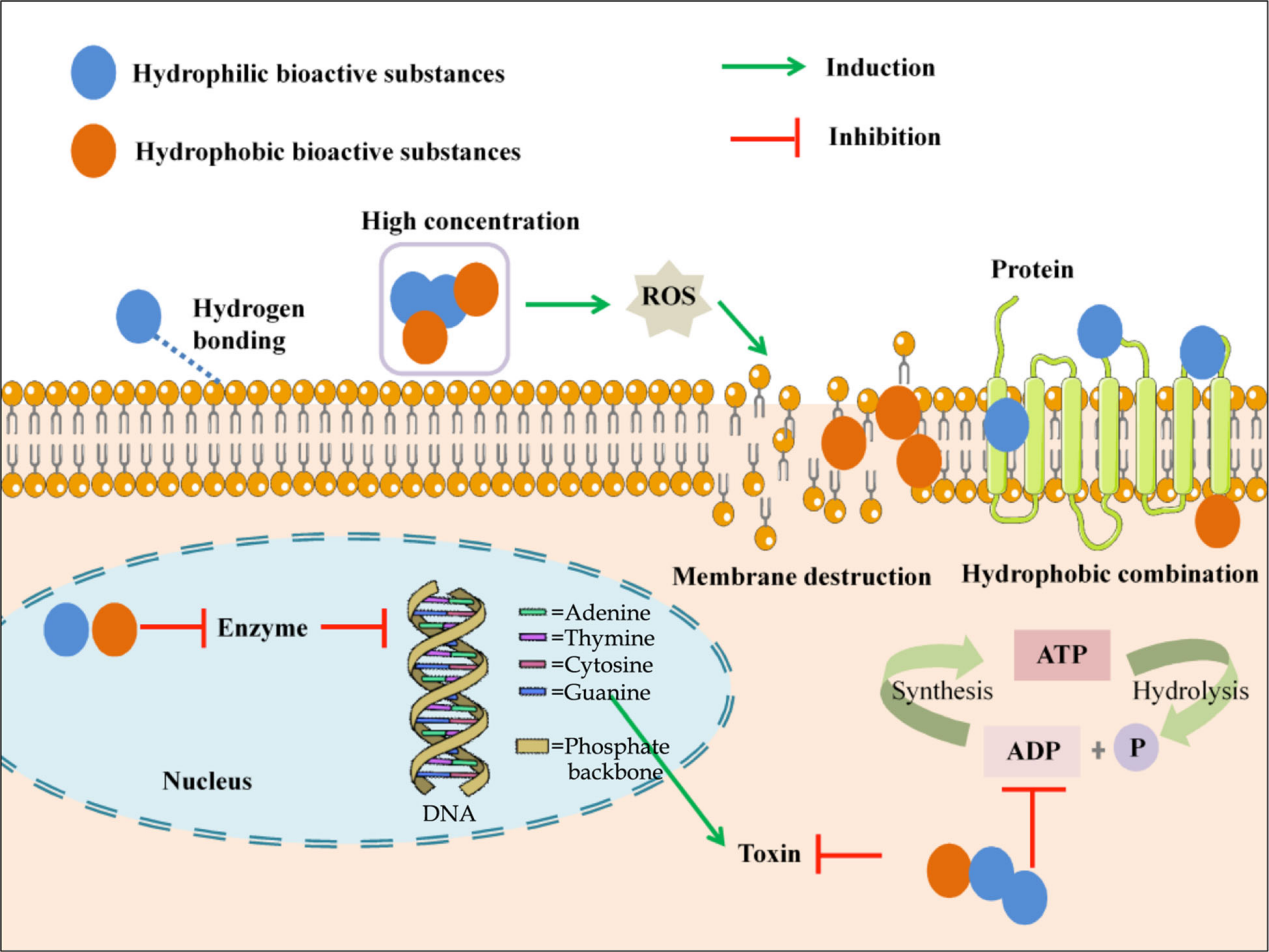
Mechanism of anti-bacterial and anti-fungal effects of natural bioactive substances

The resistance of natural substances to bacteria or fungal attack/growth is mainly due to the destruction of cell membranes. The cell membrane is responsible for respiration and transport processes, osmotic regulation, biosynthesis, and cross-linking of some essential substances (e.g. peptidoglycan, and lipids). Therefore, the destruction of the cell membrane can result in metabolic dysfunction and finally lead to bacterial death (Hartmann et al. 2010). In general, the damage of natural bioactive substances to bacterial cell membranes can follow either a direct and an indirect mechanism (Fig. 1).

Some natural substances could directly kill bacteria through interaction with the cell membrane (e.g. proteins, and lipid layers). This may occur via the destruction of cellular proteins. For example, the sugarcane bagasse extract, containing some phenolic acids proved to decrease the content of cellular soluble protein content by permeating and disrupting the cell membrane of bacteria (Zhao et al. 2015). Moreover, some of the bioactive substances could change the hydrophobic properties of the cell wall or cell membrane. For instance, phenolic acids (eg., gallic acid, and cinnamic acid) can induce irreversible changes of the membrane through altering hydrophobicity or forming local rupture/pore in the cell membranes, thus resulting in leaking intracellular compounds (Borges et al. 2013). The essential oil can cause irreversible damage to the cell membrane by inducing the lysis of bacterial cells (cytolysis), thus resulting in cell death (Villa and Veiga-Crespo 2014). The interaction of chitosan with bacterial cell surfaces results in an increase in cell membrane permeability and its destruction caused by non-specific binding of polycationic chitosan molecules (Su et al. 2009). Besides, some natural substances, such as flavonoids, can interact with lipid bilayers of bacteria through accumulating non-polar flavonoids in the hydrophobic region of the cell membrane or forming hydrogen bonds between polar head groups of lipids and hydrophilic flavonoids at the interface (Tsuchiya 2015). As an example, quercetin, rutin, and salidroside were found to decrease lipid bilayer thickness, thus disrupting the lipid monolayer structure (Sanver et al. 2016). In addition, some flavonoids can interact with phospholipids, thus resulting in structural changes in the membrane (Górniak et al. 2019). For example, catechins are able to destroy the bacterial membrane through binding to the lipid bilayer, thus inactivating or inhibiting the synthesis of intracellular and extracellular enzymes of bacteria (Reygaert 2014).

The destruction of bacterial cell membrane structures by reactive oxygen species (ROS) due to the high concentrations of bioactive substances is another mechanism of action that may be followed. For instance, catechins were found to kill bacteria or fungi through an oxidative burst by the generation of ROS which induces membrane permeability or causing membrane damage at high concentrations (Fathima and Rao 2016).

The interaction between polyphenols and bacterial cell membranes is especially related to the structure of polyphenols. To be exact, previous studies have found that the position of hydroxyl groups in polyphenols, and the presence of methoxy groups in the C ring can significantly influence their antimicrobial activities. For instance, some flavonoids with more methyl groups in the B ring proved to render stronger anti-bactericidal effects because of their lipophilic properties (Matijaševic et al. 2016).

#### Interference with nucleic acid synthesis

Some natural phytochemicals are inhibitors of nucleic acid synthesis, especially nucleic acid-related enzymes in bacteria (Fig. 1). On the one hand, natural bioactive substances affect DNA supercoiling. One route is direct interaction with the amino acid residues of DNA gyrase. For example, some flavonoids (eg., chrysin, and kaempferol) can form hydrogen bonds to occupy ATP binding pocket between its –OH groups and the B subunit of gyrase (Wu et al. 2013). Another way is that some natural substances competitively interact with the ATP binding site of the DNA gyrase B subunit according to some molecular docking results (Wu et al. 2013). Thus, these compounds combine with DNA to form a complex that eventually leads to DNA degradation, blocking cell transcription, and replication (Fang et al. 2016). On the other hand, some bioactive substances affect the rearrangement of nucleic acid double chains. The mechanism of this action is similar to that of the DNA gyrase mentioned above, which makes helicases as important targets.

Bioactive substances such as epigallocatechin gallate can affect the folic acid synthesis pathway through inhibiting dihydrofolate reductase (Raju et al. 2015). This reaction affects the synthesis of pyrimidines and purines in the bacteria, thus affecting DNA transcription and replication (Bhosle and Chandra 2016). This effect may result in the loss of bacterial organs. For example, phloretin has proven to inhibit the formation of fimbriae through influencing DNA, thus resulting in a decrease of adhesion and the break down of biofilms. This weakens the bacteria to resist antibacterial drugs (Lee et al. 2011). Chitosan can also interfere with the synthesis of DNA and mRNA in bacteria or fungi (Su et al. 2009).

#### Reducing metabolism

Natural bioactive substances regulate bacterial metabolism mainly by inhibiting electron transport chains and ATP synthesis/hydrolysis (Fig. 1). For example, quercetin, quercetin-3-glucoside, and quercetin-3-*O*-rhamnoside have proven to prevent ATP hydrolysis (Chinnam et al. 2010). Moreover, proanthocyanidins isolated from cranberries down-regulated the ATP synthesis (Ulrey et al. 2014). For some fungus, amylase and protease can be inhibited by essential oils, which stop toxin production and electron flow, thus resulting in coagulation of the cell content. A decline in metabolism will lead to the inhibition of biofilm formation, which is a kind of three-dimensional biofilms formed by mature cells (Adlard 2010). For example, anti-biofilm activity was decreased by flavonoids and triterpenes isolated from the extracts of *Ficus Sansibarica* warb (Awolola et al. 2014).

### Anti-virus mechanisms

#### Inhibition of binding to host cells

Unlike other microorganisms, with the exception of nucleic acid, viruses do not have the structures that are the same as other living cells. Viruses consist of a protein coat (a capsid), protein subunits (capsomeres), a small number of enzymes for infection of host cells, and a central core of nucleic acid (Boxman 2013). Therefore, viruses must rely on host cells to survive. Some polyphenols, polysaccharides, and proteins are thought to prevent the viral attachment to host cells, either by causing damage on the viral capsids or change of the receptors on the cell membranes (Li et al. 2013). Moreover, bioactive substances isolated from some specific plants containing terpenoids and lignoids have shown antiviral activities against severe acute respiratory syndrome coronavirus through inhibiting postbinding and entering to host cells (Wen et al. 2007). Furthermore, inhibition of viral infection by black raspberry juice (contains polyphenols) on murine norovirus-1 and feline calicivirus-F9 probably occurs at the internalization of virions into the cell or the attachment of the viral surface protein to the cellular receptors (Oh et al. 2012).

#### Destroying the virus envelope and modifying the capsid

When the virus has not yet entered the host cell, destroying the virus envelope is effective in the prevention of food-borne viruses. Several studies have found that various plant-based bioactive substances, such as essential oils, seem to act directly on enveloped viruses (Schnitzler et al. 2011). Moreover, some bioactive substances can modify the virus capsid. For instance, cranberry juice and proanthocyanidins, which contain polyphenols were found to damage the capsid of feline calicivirus (Su et al. 2010). In addition, a visible capsid disintegration of murine norovirus was found in the essential oil-treated samples (Gilling et al. 2014).

Several studies have found that some negatively charged viruses could bind positively charged chitosan, thus resulting in weakening or disruption of the capsid structure of viruses (Su et al. 2009). Moreover, various chitosans with different charges, molecule size, and solubility show different inhibition on food-borne viruses (Su et al. 2009). The difference in the effectiveness of chitosan on the reduction of foodborne viruses suggests that its use as an inhibitory agent may be limited.

However, destroying the virus envelope and modifying the capsid are ineffective against viruses once they are located within cells. Some non-enveloped viruses can protect the integrity of the viral nucleic acid and initiate infection by adsorption to the host cell (Cliver 2009). Some bioactive substances have been shown to resist food-borne viruses by inhibiting nucleic acid replication or interfering with lysosome production, which will be discussed in detail as adjuvant treatment of food-borne diseases in this review.

### Detoxification

In addition to bacteria, fungi, and food-borne viruses, food contaminants also include some toxins produced by microorganisms, and chemical residues, among others. Certain natural bioactive substances can interact with toxins or chemical residues. For example, aflatoxin B1 (AFB1), produced by the common *Aspergillus flavus* and *Aspergillus parasiticus*, is common and widespread in food products, including poultry, corn, rice, oilseeds, dried fruits, and peanuts, especially in hot, humid, and unsanitary conditions (Hamid et al. 2013). To solve this problem, chemoprevention strategies aimed at reducing AFB1 toxicity in both animal-based and plant-based food have been considered. Some studies suggest that these naturally active substances act as antioxidants, increasing the expression of many large molecules in poultry cells, such as phase II enzymes to act against aflatoxicosis (Rawal et al. 2010). Moreover, for some plant-based food, compounds from essential oil, such as cinnamaldehyde, could cause a reduction in AFB1 through structural degradation or down-regulating the concentration of ROS because of its antioxidant properties (Sun et al. 2015).

Chemical residues, such as nitrite, are ubiquitous components of dietary regimens, and can be found in cured or pickled meats (eg., bacon, fermented sausage, hot dogs, ham and smoked meat), as nitrate in vegetables (e.g., spinaches, beets, radishes, celery, and cabbages), fertilizers and polluted drinking waters. Several studies have shown that some natural bioactive substances, especially polyphenols, may interact with nitrite through oxidation or nitration directly. For example, catechins and rutin-like flavones react mainly by oxidation, whereas hydroxycinnamates react mainly by nitration. Besides, flavonols with a hydroxyl group at the 3-position reduce nitrous acid to NO (D’Ischia et al. 2011).

## Adjuvant treatment of food-borne diseases with natural bioactive substances

Many natural bioactive substances are used not only as food additives to prevent the production of food-borne viruses and food spoilage, but also have the potential in the treatment of some food-borne diseases. The following subsections provide details about their role in food.

### Detoxification of heavy metals

**Fig. 2:**
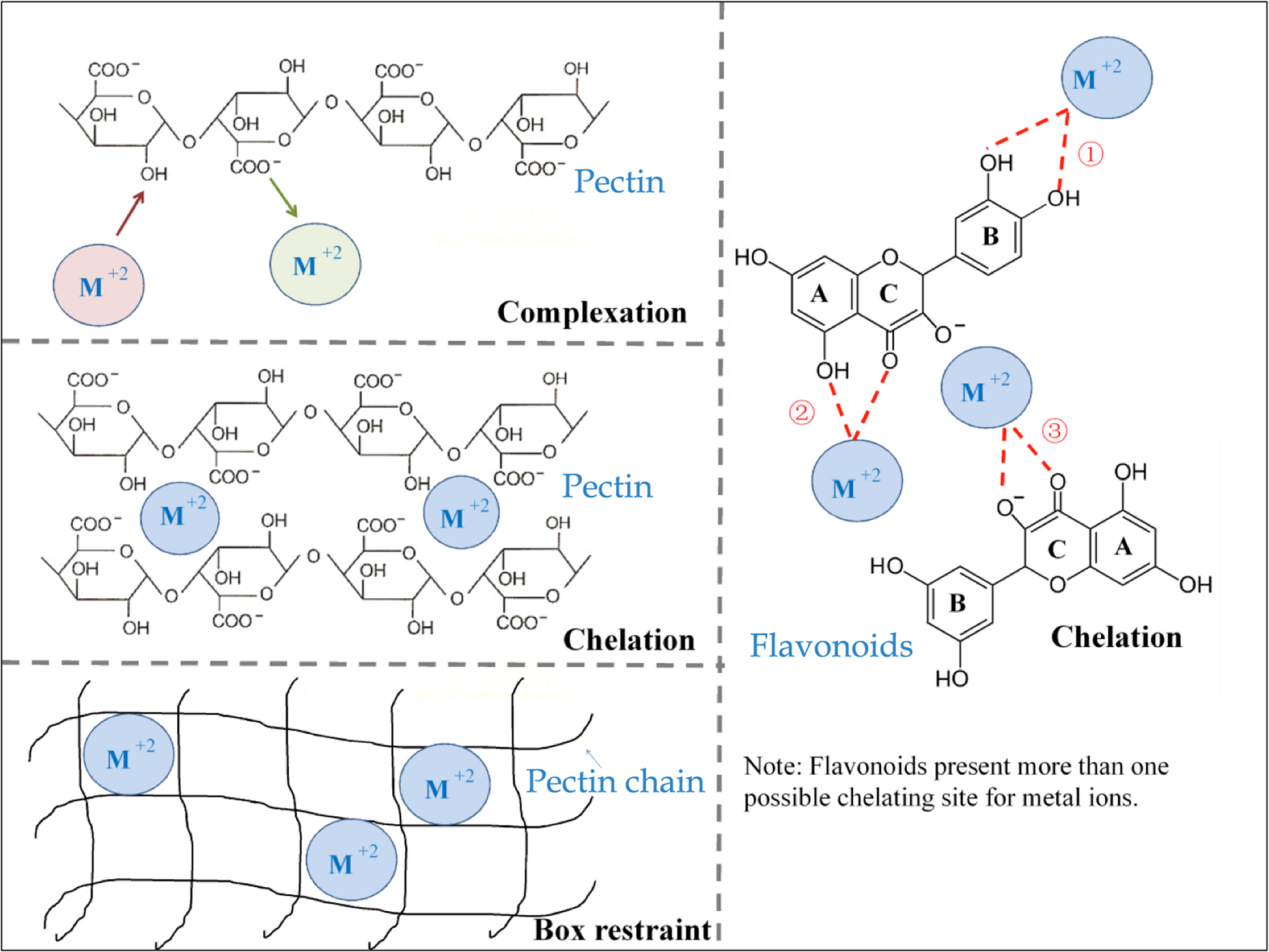
Interaction natural between bioactive substances and heavy metals

Heavy metals from the environment and food chain create a potential health hazard, thus becoming toxic when they are not metabolized and hence accumulate in the human body. Natural bioactive substances, such as flavonoids and pectin, among others, have been recognized in the disease prevention recovery against heavy metal intoxication (Sharma et al. 2016). These compounds affect biological systems not only through the chelation of toxic metal(s) but also via formatting a “box” structure to restraint the metal (Fig. 2). For example, pectin can bind with metals such as Pb, Cu, Co, Ni, Zn, and Mg, among others. Pectin is used for effective treatment against poisoning in clinical studies as a chelator (Zhao et al. 2008). Moreover, some flavonoids have been shown to chelate heavy metal ions (Fig. 2). On the other hand, pectin can form a “box” like structure between the metal ions and the ionized carboxyl groups, thus decreasing the absorption of heavy metals (Fig. 2). Complexes of some proteins with heavy metals can also reduce the absorption of heavy metals in the body (Kinoshita et al. 2013). In addition, some bioactive substances detoxify certain metal ions indirectly, for example, increasing the formation of urine in the body, thus accelerating their metabolism (Sharma et al. 2016).

### Anti-viral effects

Numerous experiments based on animal or cell models suggest that some natural bioactive substances or their extracts show anti-rotaviral effects (Gandhi et al. 2016). For example, Alfajaro et al. (2014) suggested that the *Sophora flavescens* extract is a potential curative medication for rotaviral diarrhea in pigs. Besides, *Calliandra haematocephala* leaf extracts showed anti-rotaviral effects in mice (Shaheen and Mostafa 2015). On the one hand, this antiviral effect was shown directly earlier in this review. The natural bioactive compounds interact directly with viruses and cause irreversible damage or reversible blocking of certain regions/areas (Howell and D’Souza 2013). In addition, some natural bioactive compounds prevent viruses from host cell binding and/or entry into the host (D’Souza 2014). For instance, the specific binding ability of human NoVs to salivary HBGA receptors could effectively be enhanced or decreased by fruit and vegetable extracts (Jacob et al. 2014). Moreover, natural bioactive substances interfere with DNA replication or inhibit viral antigen secretion after the virus entering the host cell (Li et al. 2018). In addition, natural bioactive substances may improve immunity in the host. For example, bioactive substances from ginger and garlic showed immune promoting effects that prevented the human body from infection (Pandey 2018).

### Anti-bacterial and anti-fungal effects

The growing problem of antibiotic resistance has made the routine therapy of many bacterial and fungal infectious diseases challenging (Hare 2009). Therefore, considering that the microbial resistance has become an increasing global problem, there is a need to find new potent antimicrobial agents as accessories to antibiotic therapy. Several natural bioactive compounds not only have been tested to combat resistant bacteria (as discussed earlier) but also show a reduction of multidrug resistance as an efflux pump inhibitor in bacteria (Kuete et al. 2011). On the one hand, many natural active substances can inhibit the growth of some pathogenic microorganisms or the formation of biofilm in the human body. For example, the consumption of green tea polyphenols shows antimicrobial activity and the inhibition of biofilm formation in the human oral cavity (Cho et al. 2010). However, after digestion through the gastrointestinal tract, the direct antibacterial properties of some natural bioactive substances, such as rose phenolic extracts are greatly weakened, which is mostly due to the chemical effects such as pH (Zhang et al. 2016a, b). Therefore, several studies have focused on nanoparticles against bacterial gastrointestinal pathogens that contained chitosan nanoparticles loaded with phenolic compounds (Madureira et al. 2015). Furthermore, several research reports have suggested that certain natural bioactive substances should be considered as a strategy to defend against fungi or bacteria; these could be used in combination with antibiotics to provide synergistic effects (Coutinho et al. 2009; Coutinho et al. 2008). The main mechanism involved in this application is increasing the sensitivity of the bacteria to antibiotics. For example, phenolic-rich maple syrup extracts were found to inhibit efflux pump activity as well as significantly repressing multiple-drug resistance genes, thus enhancing bacterial antibiotic susceptibility (Maisuria et al. 2015). Moreover, thyme essential oil was found to inhibit multidrug-resistance of some food-borne bacterial strains (eg., *Staphylococcus*, *Enterococcus*, and *Escherichia*) (Sienkiewicz et al. 2012). In addition, most natural bioactive substances have antioxidant and anti-inflammatory effects. Therefore, they are used in the treatment of many fungal or bacterial toxins or induced inflammation (Palaska et al. 2013; Iranshahi et al. 2015).

## Conclusion

Several natural bioactive substances, including polyphenols, proteins, essential oils, and polysaccharides, or their extracts, have demonstrated anti-virual, anti-bacterial, and anti-fungal effects as well as inhibiting the adverse effects of containments. Therefore, these natural bioactive substances are widely used in agricultural farming, animal husbandry, and food processing as natural medicinal products or food additives. The advantages of natural bioactive substances in controlling food-borne viruses and contaminants include their generally mild treatment condition compared to the traditional physical and chemical treatments. These natural bioactive substances show inhibition of microorganisms by destroying the cell membrane, interfering with nucleic acid synthesis, and reducing the metabolism of bacteria and fungi. They also show inhibition of binding to host cells, destroying the envelope, and modifying the capsid on the virus. In addition, these natural bioactive substances were found to have detoxification ability against food poisoning caused by heavy metals in the body. They could also have a therapeutic effect on some diseases caused by viruses, bacteria, and fungi. Due to these properties, natural active substances are not only used in the field of food production and processing but also play an important role in the treatment of foodborne diseases. Research on the therapeutic effects of natural active substances on food-borne diseases is one of the fields that deserve particular future attention.

## Data Availability

Not applicable.
